# The influence of host genetics on erythrocytes and malaria infection: is there therapeutic potential?

**DOI:** 10.1186/s12936-015-0809-x

**Published:** 2015-07-29

**Authors:** Patrick M Lelliott, Brendan J McMorran, Simon J Foote, Gaetan Burgio

**Affiliations:** John Curtin School of Medical Research, Australian National University, Canberra, ACT Australia

**Keywords:** Malaria, *Plasmodium*, Host, Polymorphism, Erythrocyte, Red blood cell, Invasion, Growth, Cytoadherence, Phagocytosis

## Abstract

As parasites, *Plasmodium* species depend upon their host for survival. During the blood stage of their life-cycle parasites invade and reside within erythrocytes, commandeering host proteins and resources towards their own ends, and dramatically transforming the host cell. Parasites aptly avoid immune detection by minimizing the exposure of parasite proteins and removing themselves from circulation through cytoadherence. Erythrocytic disorders brought on by host genetic mutations can interfere with one or more of these processes, thereby providing a measure of protection against malaria to the host. This review summarizes recent findings regarding the mechanistic aspects of this protection, as mediated through the parasites interaction with abnormal erythrocytes. These novel findings include the reliance of the parasite on the host enzyme ferrochelatase, and the discovery of basigin and CD55 as obligate erythrocyte receptors for parasite invasion. The elucidation of these naturally occurring malaria resistance mechanisms is increasing the understanding of the host-parasite interaction, and as discussed below, is providing new insights into the development of therapies to prevent this disease.

## Background

The clinical symptoms of malaria occur during the blood stage of *Plasmodium’s* life cycle. Safely ensconced within the host erythrocyte, parasites develop and replicate whilst concealing their presence from the immune system. After consuming the contents of the host cell, and growing and multiplying to fill the available space, the progeny egress as merozoites, and, after briefly existing extracellularly, invade fresh erythrocytes, continuing the cycle of growth and proliferation. This cycle depends on an elaborate interplay between host and parasite proteins, which has been meticulously established over thousands of years of co-evolution. As such, any perturbations to the composition or arrangement of proteins in the host erythrocyte can potentially impede the parasite’s growth and survival, and thereby increase the resistance of the host to infection. Indeed, abnormalities of the erythrocyte are relatively common, especially in populations residing in malaria-endemic regions, consistent with the positive selection for these conditions. The mechanistic basis for protection against malaria is partly understood in some abnormalities; parasite invasion and intraerythrocytic development are often affected. However, recent studies have revealed a more complex picture, with many conditions sharing multiple and over-lapping pathways that advantage the host. The understanding of the parasite-erythrocyte interaction is also being challenged as novel and highly intimate relationships between the parasite and its host cell are discovered. This review will summarize the current knowledge regarding *Plasmodium’s* interaction with abnormal host erythrocytes, the mechanisms by which these abnormalities can inhibit the blood stage of *Plasmodium’s* life cycle, and the implications of these findings for malaria treatment.

## Genetic erythrocyte abnormalities and malaria susceptibility

Erythrocytes have a limited lifespan (120 days in humans) and, therefore, must be continually replenished in a process known as erythropoiesis. In this process haematopoietic stem cells replicate and differentiate into erythroblasts, and following expulsion of their nucleus and most organelles, develop into reticulocytes. Reticulocytes are released from the bone marrow into the bloodstream and following further depletion of organelles and intracellular RNA, become mature erythrocytes. There are a number of erythrocyte disorders that result from mutations in the genes expressed during erythropoiesis; many are highly prevalent, particularly in populations with a long history of malaria exposure. It was first observed nearly 70 years ago that people with ‘sickled’ erythrocytes were less likely to suffer from malaria [1]. This condition is common in various West and Central African ethnicities. Now known as sickle cell trait, this and many other erythrocytic disorders, have been strongly associated with reduced malaria susceptibility. In fact, mutations causing erythrocyte abnormalities are the most commonly observed genetic traits in humans [2]. Genetic mutations associated with malaria resistance have been extensively reviewed previously [3–5]; a summary of known erythrocytic genetic disorders and their association with malaria susceptibility is given in Table 1.

**Table 1:** Erythrocyte disorders and the possible mechanisms by which they protect against malaria

Erythrocyte disorder	Malaria susceptibility	Invasion	Growth	Cytoadherence	Erythrocyte senescence	References
Sickle cell trait (HbAS)	↓	↓ (Low oxygen) or normal	↓ (Low oxygen)	↓	↑	[61, 68–70, 104, 122, 125]
Sickle cell disease (HbSS)	↓	↓ (Low oxygen) or ↑	↓ (Low oxygen)	↓	Unknown	[61, 70, 104]
HbAC	↓	Normal	↓	↓	Unknown	[68, 69, 105]
HbCC	↓	Normal	↓	↓	Unknown	[61–63, 69, 105]
HbAE	↓	↓	Normal	Unknown	Unknown	[178]
HbEE	Unclear	Normal	↓ or normal	Unknown	Unknown	[64]
α^+^ thalassaemia (−α/αα)	↓	Normal	↓ (High oxygen)	↓	Normal	[27, 65, 106]
α^0^ thalassaemia (−/αα)	↓	↓ or normal	↓ or normal	↓	Unknown	[106]
HbH thalassaemia (−/−α)	↓	↓ or normal	↓	↓	↑	[66, 67, 106, 122]
β^0^ thalassaemia (−/β)	Unclear	Normal	↓ (High oxygen)	Unknown	↑	[65, 122]
G6PD deficiency	↓	Normal	↓or normal	Unknown	↑	[3, 129–131]
PK deficiency	Unknown	↓	Normal	Unknown	↑	[133]
Duffy negativity	↓ (In *P. vivax*)	↓ (in *P. vivax*)	Normal	Unknown	Unknown	[9–11]
ABO blood group	↓ (O type) ↑ (A and AB type)	Normal	Normal	Unknown	↑ (For O type)	[135, 136]
CR1 deficiency	Unclear	↓	Unknown	Unknown	Unknown	[25–27]
GYPA deficiency	Unknown	↓	Unknown	Unknown	Unknown	[17, 18, 20]
GYPB deficiency	Unknown	↓	Unknown	Unknown	Unknown	[21, 24]
GYPC deficiency	Unclear	↓	Unknown	Unknown	Unknown	[23]
Basigin deficiency	Unknown	↓	Unknown	Unknown	Unknown	[28]
CD55 deficiency	Unknown	↓	Unknown	Unknown	Unknown	[31]
Southeast Asian ovalocytosis (SAO)	↓	↓	Normal	↑	Unknown	[37, 45, 48, 49, 51, 107]
Hereditary elliptocytosis	Unknown	↓ or normal	↓	Unknown	Unknown	[54, 58, 59]
Hereditary spherocytosis	Unknown	↓ or normal	↓	Unknown	Unknown	[57, 59]
Erythropoietic protoporphyria	Unknown	Unknown	↓	Unknown	Unknown	[84]

↓ Decreased, ↑increased. “Malaria susceptibility” refers to evidence for an association with reduced risk of severe or uncomplicated malaria. “Growth” refers to the ability of an individual parasite to replicate and egress from the erythrocyte. Studies performed with *P. falciparum* unless otherwise indicated.

## Mechanisms by which erythrocyte abnormalities protect against malaria

Early studies towards identifying malaria protective mechanisms imparted by erythrocyte abnormalities largely focussed on the ability of the parasite to invade and grow within erythrocytes. These studies were facilitated by an in vitro culturing system for *Plasmodium falciparum*, which allowed researchers to easily compare between erythrocytes obtained from subjects with various genetic conditions. As the understanding of malaria pathogenesis was expanded, the importance of factors such as cytoadherence and the ability of the immune system to detect parasitized erythrocytes was also recognized. Studies then addressed how erythrocyte abnormalities could affect not only parasite invasion and growth under in vitro conditions, but also their effects in vivo, and how this may influence host resistance to malaria infection. This review considers how specific erythrocyte abnormalities affect four distinct features of the parasite-host interaction. Namely, merozoite invasion, parasite growth, cytoadherence, and erythrocyte senescence.

### Merozoite invasion of the erythrocyte

The brief interval between parasite egress and reinvasion of a new erythrocyte has long been considered a potential weak point in its blood stage lifecycle. In vitro studies have shown that *P. falciparum* merozoites are exposed for approximately 2 min from egress to reinvasion, while the actual invasion event is completed in less than 30 s [6]. During this period the parasite is particularly susceptible to host recognition and attack mechanisms, due to the potential for direct contact with opsonins and immune cells; the parasite also becomes nonviable if it fails to invade a new cell [7]. Consequently, any disruption to the invasion pathway is beneficial to the host, and several abnormalities are known to directly affect this process.

There are several well-characterized genetic mutations in erythrocyte membrane proteins that are associated with impaired merozoite invasion. For example, African populations carry a highly prevalent single nucleotide polymorphism (SNP) in the promoter region controlling expression of the Duffy antigen receptor for chemokines gene (DARC or Duffy; rs2814778). The SNP prevents erythrocytic expression and results in the Duffy-negative blood phenotype [8]. Duffy is a ligand for a *P. vivax* merozoite protein called the Duffy-binding protein (PvDBP) and the Duffy-PvDBP interaction is essential for merozoite invasion of the erythrocyte [9, 10]. Therefore, Duffy-negative individuals are protected against *Plasmodium vivax* infection and in areas of Africa with a high incidence of this SNP, *P. vivax* infection is virtually non-existent [11, 12]. In contrast *P. vivax* is common in other malaria endemic areas where populations do not carry this polymorphism (Asia and South America) [12, 13]. However, complete protection due to this polymorphism has been questioned recently by observations of *P. vivax* infection in Duffy negative individuals in several studies [14–16]. In contrast to *P. vivax*, *P. falciparum* merozoites utilize several redundant invasion pathways and different host ligands. In 1977, Miller et al. [17] reported that erythrocytes from individuals with the rare En (a−) mutation were resistant to *P. falciparum* merozoite invasion. En (a−) erythrocytes lack Glycophorin A (GYPA), highlighting a possible role for this protein as a parasite ligand. Indeed, GYPA is now known to be a binding partner of *P. falciparum* erythrocyte binding antigen 175 (PfEBA-175) [18, 19], and more recently, was found to bind to merozoite surface protein 1 (PfMSP1) [20]. In the later study, the erythrocyte surface band 3-GYPA complex was shown to be essential for invasion by both *P. falciparum* and the rodent malaria parasite, *Plasmodium yoelii*. Mutations in other erythrocyte glycophorins (GYPB and GYPC) are common in endemic malaria populations, and recent studies have demonstrated comparable importance for these proteins in parasite invasion. The *P. falciparum* protein erythrocyte-binding ligand-1 (PfEBL-1) binds to GYPB [21, 22], while GYPC is a receptor for erythrocyte binding antigen 140 (PfEBA-140) [23]. Antibodies against the binding domains of both PfEBL-1 and PfEBA-140 inhibit merozoite invasion, while erythrocytes deficient in either GYPB (blood group S-s-U-), or GYPC (Gerbich negativity) are resistant to invasion [21–24]. Erythrocyte expressed complement receptor 1 (CR1) is also a demonstrated receptor for the *P. falciparum* merozoite protein PfRh4, with antibodies against CR1, as well as soluble CR1 protein, preventing invasion [25, 26]. This may partially explain the enhanced malaria resistance exhibited by individuals with inherited mutations resulting in CR1 deficiency [27]. Recently, two high throughput screening approaches were developed to identify novel host binding partners of merozoite proteins involved in invasion. The first utilized the Avidity-based extracellular interaction screen (AVEXIS) to identify binding interactions between erythrocyte and merozoite proteins [28]. This led to the discovery of a single definitive interaction between the host protein basigin and *P. falciparum* reticulocyte-binding protein homologue 5 (PfRH5). Antibody blocking studies demonstrated that this interaction is essential for invasion by all *P. falciparum* strains tested to date [28, 29], and erythrocytes expressing naturally occurring mutations in basigin (known as the Ok blood group antigen) also prevented parasite invasion [28]. This host-parasite receptor ligand interaction also appears to be important in clinical disease based on the findings that naturally acquired antibodies to PfRH5 are associated with improved outcomes to infection [30]. Another screening approach has utilized in vitro differentiated human erythroid cells in conjunction with short hairpin RNA (shRNA) libraries to target and screen for erythroid-expressed proteins necessary for *P. falciparum* merozoite invasion. As well as corroborating a requirement for basigin and CR1 in invasion, a novel interaction involving erythrocyte CD55, also known as Decay accelerating factor (DAF), was found to be essential for the invasion process. The requirement for this interaction was confirmed utilizing naturally-occurring CD55 null erythrocytes, which were refractory to invasion by several *P. falciparum* strains, including clinical isolates [31]. Interestingly, polymorphisms in CD55 are enriched in populations with historical exposure to malaria [32, 33], indicating a possible selection pressure on this gene. Importantly, the identification of host cell receptor-parasite ligand interactions has produced several possible targets for vaccine or therapeutic development.

The erythrocyte membrane is supported by an underlying network of proteins called the cytoskeleton, which provides the cell with structure and deformability, and serves as an anchoring point for membrane proteins. Hereditary elliptocytosis and hereditary spherocytosis are conditions caused by mutations in genes encoding cytoskeletal proteins, including alpha spectrin, beta spectrin, ankyrin, band 3 and protein 4.1, which affect molecule conformation or abundance. Mutations in genes encoding some of these proteins have been associated with increased protection against infection or severe forms of malaria. A well-known example is Southeast Asian ovalocytosis (SAO), which is caused by mutations in the gene encoding band 3. Band 3 is a transmembrane protein that normally exists as a dimer or tetramer and interacts with both the cytoskeleton and cell membrane; it also functions as an anion transporter. In SAO, mutations disrupt the anion transporter activity and result in the formation of large-sized aggregates [34–36], resulting in increased cell membrane rigidity [37–39], oval shaped cell morphology, reduced band 3 mobility in the membrane [40–42], and decreased expression of surface antigens [43, 44]. Homozygous inheritance of SAO-causing band 3 mutations results in embryonic lethality, however heterozygosity is associated with a marked protection against severe *P. falciparum* malaria, particularly cerebral malaria [45, 46]. Despite the fitness cost, frequencies of these mutations are remarkably high some populations, particularly those residing in Papua New Guinea [47]. There is strong evidence that *P. falciparum* merozoite invasion of SAO erythrocytes is impaired, which could plausibly explain the reduced malaria susceptibility conveyed by this condition [37, 39, 48, 49]. Mechanistic explanations variously include increased membrane rigidity, direct interference with parasite/band 3 binding [37–39, 50, 51], a loss or reduction of merozoite ligands, such as GYPA (which forms a complex with band 3) [20, 43, 44], and reduced band 3 mobility [52, 53]. Interestingly, cells with artificially inhibited band 3 mobility also exhibit reduced rates of merozoite invasion [53]. Erythrocytes from patients with other forms of hereditary elliptocytosis and spherocytosis have shown more variable, and sometimes inconsistent effects on parasite invasion. Erythrocytes deficient in protein 4.1 are reported to be less vulnerable to parasite invasion [54], which could possibly be explained by a secondary deficiency of GYPC (90% less in protein 4.1 deficient cells), although the role of GYPC as a parasite ligand was not investigated in this study. Utilizing a recently developed method for measuring merozoite invasion in vivo [55, 56], a study in mice carrying a mutation in ankyrin-1 (*Ank1*) reported reduced rates of erythrocyte invasion by *Plasmodium chabaudi* merozoites; this coincides with increased resistance and reduced parasitaemia reported in these mice [57]. Investigations of hereditary spherocytosis caused by mutations in alpha spectrin have also reported reduced merozoite invasion [58], however these studies are contradictory and the exact effects of these conditions remain inconclusive [59].

Overall, the disruption of parasite invasion is an obvious and testable mechanism by which erythrocyte disorders can protect the host against malaria infection. This is most evident for mutations that disrupt a specific parasite-erythrocyte binding interaction. Erythrocytes with structural abnormalities, such as spherocytosis and ovalocytosis, may also present the merozoite with a less than ideal substrate for invasion, however the mechanisms are difficult to establish, and findings are less consistent.

### Intraerythrocytic growth

After gaining entry into an erythrocyte the *Plasmodium* parasite must dramatically remodel the host cell cytoplasm and membrane in order to grow, replicate, and eventually egress. The success of the parasite at this stage depends on several factors; the ability to digest haemoglobin, establishment of pathways to import nutrients, export waste products and traffic synthesized proteins around the cell, scavenging of host proteins, and, finally, effective dissolution of the cell membrane during egress.

There are five types of haemoglobinopathies found at high frequencies in both extant and historically malaria afflicted populations, and each has been implicated to varying degrees in perturbing the intraerythrocytic development and growth of *Plasmodium*. Three of these involve single amino acid substitutions in the beta globin chain of haemoglobin; sickle cell (HbS, β6Glu to Val, rs334), haemoglobin C (HbC, β6Glu to Lys, rs33930165), and haemoglobin E (HbE, β26Glu to Lys, rs33950507). The remaining two types of haemoglobinopathies, alpha and beta thalassaemia, arise due to reduced expression of the alpha and beta globin chains of haemoglobin, respectively, caused by various polymorphisms in the genes encoding these proteins. Impaired parasite growth in these cells (here defined specifically as the ability of an individual parasite to grow, replicate and produce new viable merozoites) has been frequently reported, although studies are often contradictory (for review see Taylor et al. [60]). An impairment in parasite growth has been consistently observed in homozygous haemoglobin C (HbCC) erythrocytes [61–63], which is proposed to be caused by spontaneous degradation of parasites in a subset of damaged mutant cells with an increased haemoglobin concentration [63]. Parasite growth is also impaired in homozygous haemoglobin E (HbEE) erythrocytes [64]. Two early studies by Friedman et al. [61, 65] indicated a role for oxygen tension in parasite growth in heterozygous HbS (HbAS or sickle cell trait), homozygous HbS (HbSS or sickle cell disease), alpha thalassaemia trait, and beta thalassaemia minor. Under low oxygen tension parasitized HbAS and HbSS cells underwent sickling and growth was attenuated. Conversely, in thalassaemic cells growth impairment occurred only under high oxygen tension. In the more severe form of alpha thalassaemia, known as haemoglobin H (HbH), a growth impairment has been consistently observed, however the mechanism remains unknown [66, 67]. More recent studies have suggested two novel mechanisms of parasite growth impairment in haemoglobinopathies. A study by Cyrklaff et al. [68] describes a process whereby host actin filaments are utilized by the parasite in developing Maurer’s clefts. In HbAS and heterozygous haemoglobin C (HbAC) cells, host actin filaments are dispersed further into the erythrocyte cytosol and are significantly longer than in normal cells, consequently, during remodelling by the parasite they fail to support the formation of normal Maurer’s clefts. The authors suggest this may be due to interference from oxidized forms of haemoglobin present in these cells, known as haemichromes. More recently, studies using a conditional protein export system indicated that the trafficking of parasite proteins across the parasitophorous vacuole is delayed and occurs at a slower rate in HbAS, HbAC, and HbCC erythrocytes, leading to reduced levels of parasite proteins in the erythrocyte cytosol and membrane [69]. Another study by La Monte et al. [70] proposes an entirely different mechanism whereby specific host micro RNAs, which are more abundant in HbAS and HbSS cells, are translocated into the parasite and fuse with parasite mRNA, inhibiting normal translation of parasite proteins. While the potential role of parasite protein trafficking and micro RNA in HbE and thalassaemia has not been investigated, it seems quite plausible that these may be a common mechanism explaining the reduced malaria susceptibility provided by these erythrocyte disorders.

*Plasmodium falciparum* growth is also impaired in spherocytotic and elliptocytotic erythrocytes. Schulman et al. [59] report that the degree of growth impairment correlates with the extent of spectrin deficiency or spectrin dimer content in spherocytotic and elliptocytotic erythrocytes respectively. This growth inhibition was not due to metabolic effects or haemolysis, and instead seems to be caused exclusively by the abnormal cytoskeleton. These results were supported by a later study, where reduced parasite growth in elliptocytotic erythrocytes was observed [54]. Finally, mice carrying a heterozygous mutation in *Ank1*, which results in severe spherocytosis in homozygotes, have an increase in terminal deoxynucleotidyl transferase dUTP nick end labelling (TUNEL) of intraerythrocytic parasites [57]. TUNEL staining indicates the presence of fragmented DNA, which has been associated with reduced growth and is likely an indication of parasite death [71]. Growth impairment in these conditions could be due to disruptions in interactions between parasite proteins and the host cytoskeleton, several of which have been well documented [72–76].

While haem biosynthesis only occurs in erythroid progenitor cells, small amounts of the enzymes used in the biosynthetic pathway remain in the mature erythrocyte [77, 78]. It has been hypothesized that the parasite may hijack these enzymes for its own haem synthesis, a notion driven by the fact that the parasite haem biosynthesis pathway is dispensable [79, 80], and that a number of these host enzymes are imported into the parasite during growth [81–83]. A recent study of ferrochelatase (Fech), which catalyses the final step in haem biosynthesis, indicates the parasite may largely depend on the erythrocyte enzyme. Fech-deficient mice (homozygous for a ferrochelatase knockdown mutation) were more resistant to *P. chabaudi* infection with parasite growth reduced, while *P. falciparum* growth was significantly impaired in cultures using Fech deficient-erythrocytes from patients with erythropoietic protoporphyria. Furthermore, erythrocytes in which ferrochelatase was inhibited pharmacologically also impeded parasite growth [84]. Another line of evidence indicates that the parasite utilizes the host kinases PAK-1 and MEK-1 to facilitate growth. They are specifically activated in infected erythrocytes and highly specific PAK-1 and MEK-1 inhibitors are potent inhibitors of parasite growth [85]. A different study has demonstrated a requirement for erythrocyte calpain-1 during parasite egress. Depletion or pharmaceutical inhibition of the enzyme prevented this stage of the parasite growth cycle [86]. Calpain-1 activation is likely initiated through a host signalling pathway involving protein kinase C (PKC). It is postulated that parasite GPCR ligands overstimulate host GPCRs, leading to G-protein alpha-q mediated activation of PKC, which in turn compromises the erythrocyte cytoskeleton through the phosphorylation of adducin and activation of the cation channel TRPC6. Rapid calcium influx through TRPC6 leads to the activation of calpain-1, which facilitates breakdown of the cell membrane and parasite egress. In support of a central role of PKC in this process, inhibitors of PKC show strong antimalarial activity during in vivo rodent malaria infection [87]. Finally, while not directly proven to be required for parasite growth, the host antioxidant enzymes superoxide dismutase [88] and peroxiredoxin-2 [89] are imported into the parasite and they retain their biological activity. In the case of peroxiredoxin-2, more than half of the parasite’s peroxide degradation capacity was estimated to derive from the host enzyme [89].

### Cytoadherence and splenic clearance of parasitized erythrocytes

The *Plasmodium* parasite distinguishes itself from most other pathogens in that its target cell, at least in mammals, is enucleate and lacks the intracellular pathogen sensing machinery present in most other cells of the body. The host must therefore rely on immune mechanisms that allow extracellular recognition (and removal) of parasitized cells.

The spleen is responsible for the removal of abnormal and senescent erythrocytes, and is also a major component in the host recognition and clearance of parasitized cells [90]. It is a highly vascularized organ comprising of inter-endothelial slits through which all erythrocytes in circulation pass (on average once every 100–200 min) [90, 91]. Erythrocytes not sufficiently deformable to pass through the slits are retained and are phagocytized by macrophages [92–94]. As the *Plasmodium* parasite grows within the erythrocyte, the cell deformability decreases [95–98], so that it may similarly be retained by the spleen and removed; even relatively immature ring-stage parasitized cells undergo an appreciable degree of splenic retention [96, 99]. However, parasites avoid splenic retention by expressing molecules that promote cell adhesion, both to other erythrocytes (called rosetting) and to endothelial cells. This results in the so-called sequestering of parasitized cells within microvasculature tissue beds, where they can continue to grow and replicate in relative isolation. In the case of *P. falciparum*, the adhesive properties are largely imparted by the interaction of PfEMP proteins with endothelial cell surface molecules such as ICAM1 and CD36 [100] and chondroitin sulphate in the placental vasculature [101]. Although the pathological consequences of *P. falciparum* sequestration are still debated (for review see Rowe et al. [102]), binding of infected cells to the endothelium has been demonstrated to promote coagulation, endothelial dysfunction, and inflammation, ultimately causing blood vessel obstruction, while sequestration in the brain microvasculature is linked to the life threatening cerebral malaria syndrome [103].

Several studies have shown that *P. falciparum* parasitized HbAS [104] [69], HbSS [104], HbAC [105] [69], HbCC [105] [69], and alpha thalassaemic [106] erythrocytes do not bind as readily to human microvascular endothelial cells in vitro (compared with parasitized HbAA erythrocytes). It was found that parasitized HbAC and HbCC erythrocytes had reduced levels of surface PfEMP-1 [105], despite displaying a normal amount of another parasite-expressed membrane associated protein; knob-associated histidine-rich protein (KHARP) [63]. Additional molecular studies indicate that parasite protein trafficking across the parasitophorous vacuole is impaired within HbAC and HbAS cells, and parasites growing within these cells form aberrant Maurer’s clefts. Together, these impairments may inhibit PfEMP-1 trafficking to the erythrocyte membrane, and explain the reduced cytoadherence of these cells [68, 69].

In contrast, SAO parasitized erythrocytes have been shown to display an increased affinity for the endothelial host receptor CD36 under flow conditions [107]. Although this seems contradictory to the known protective effect of SAO during severe malaria, it is worth noting that CD36 is an important immune cell receptor, and has been implicated in the binding and removal of parasitized erythrocytes by both platelets [108] and phagocytic cells [109]. The exact effects of increased CD36 affinity in determining resistance to malaria in SAO remain to be determined.

Erythrocyte disorders that modify the cytoadherent properties of infected cells may result in not only increased immune cell recognition, but also advantageously reduce blood vessel obstruction, coagulation, and inflammation. While good evidence implicates this protective mechanism in the aforementioned disorders, investigations of other erythrocyte abnormalities associated with malaria resistance remain to be undertaken.

### Erythrocyte senescence

Erythrocytes undergo several age-related physiological changes during their normal lifespan, which eventually trigger their phagocytosis and recycling by macrophages. This senescence is characterized by cell shrinkage, increased density and rigidity, and changes to erythrocyte surface proteins. Two major models for erythrocyte senescence are commonly reported in the literature. In the first model, here referred to as “band 3 senescence”, a build-up of haemichromes (products of haemoglobin degradation) results in crosslinking between cytoplasmic domains of band 3 proteins and formation of aggregates [110, 111]. Aggregation is facilitated by phosphorylation of band 3, another hallmark of senescence, which reduces its affinity for the cytoskeletal protein, ankyrin, and increases its mobility in the membrane [112]. Aggregated band 3 is recognized by naturally occurring antibodies, which in turn promotes complement protein C3 binding and activation [111]. This finally results in complement-mediated phagocytosis of the senescent cell [113]. The second model, referred to here as “eryptosis”, is characterized by increased intracellular calcium, activation of proteases and phosphatidylserine (PS) exposure on the external surface of the plasma membrane; the term eryptosis recognizes that these features are shared with apoptosis in nucleated mammalian cells [114]. PS exposure leads to phagocytosis of the eryptotic cell [115]. Both eryptosis and band 3 senescence are elevated in parasitized erythrocytes [93, 116–118], and in direct correlation with increasing parasite maturation [116, 119]. An increased oxidative burden imparted on the cell by the parasite is believed to be a central cause of these phenomena [116, 119]. Naturally occurring antibodies to band 3 are associated with improved malaria outcome, indicating that accelerated band 3 senescence may be an important contributor to host defence [120].

In several of the haemoglobinpathies, aberrant haemoglobin is thought to contribute towards the build-up of haemichromes in erythrocytes, thereby accelerating band 3 aggregation [121]. In ring stage infected erythrocytes, senescence of HbAS, HbH, and beta thalassaemic cells occurs more rapidly than equivalently infected normal erythrocytes [122]. This renders ring stage parasites within these cells more susceptible to phagocytosis, which could contribute toward the resistance phenotype conveyed by these disorders. Eryptosis is also enhanced in HbAS and beta thalassaemia [123, 124], and contributes to the early clearance of parasitized cells through PS exposure and phagocytosis [125].

Glucose-6-phosphate dehydrogenase (G6PDH) is a key enzyme involved in controlling oxidative stress in the erythrocyte. Polymorphisms in the X-linked G6PDH gene that result in reduced enzyme activity are relatively frequent in malaria afflicted populations, and there is good evidence that these variants are associated with a decreased risk of severe malaria [3, 126–128]. While early investigations on the potential protective mechanism reported that parasite growth is impaired within G6PD deficient erythrocytes [129, 130], this was not supported by more recent studies [131]. Instead, it seems more likely that elevated oxidative stress in the erythrocyte results in increased haemichrome formation, which results in an increased susceptibility of ring stage parasitized cells to senescence and phagocytosis [131]. G6PD deficient erythrocytes are also more susceptible to eryptosis, which may be an additional factor mediating their early clearance when parasitized [124]. Genetic-based deficiencies of pyruvate kinase (PK), a glycolytic enzyme involved in the production of ATP, have been associated with reduced susceptibility to both rodent and human malaria [132, 133]. Similar to G6PD deficiency, increased oxidative stress in these defective erythrocytes is thought to accelerate ring stage erythrocyte senescence, resulting in enhanced phagocytic clearance of parasites [134]. It has also been recently reported that parasitized O-blood group erythrocytes display increased susceptibility to band 3 senescence and phagocytosis, indicating that this mechanism may be responsible for the increased malaria resistance that has been observed for people of O-blood type [135, 136].

### Integrated mechanisms

**Figure 1:**
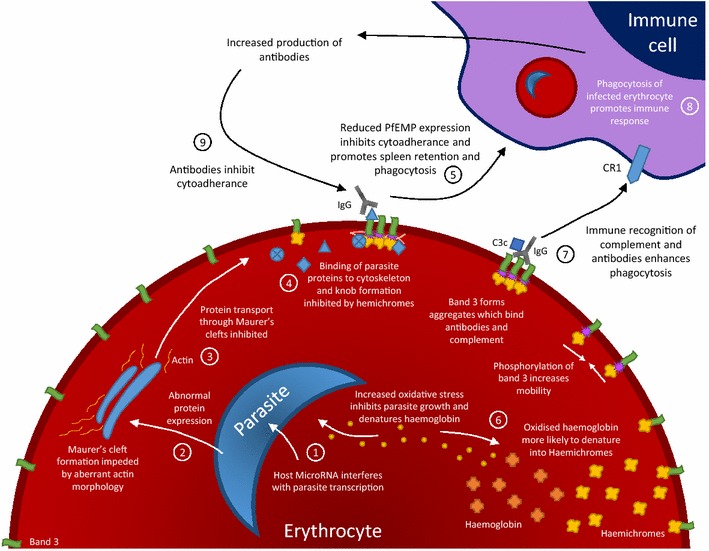
Multiple mechanisms of resistance in sickle cell trait (HbAS). Increased oxidative stress and host microRNAs in HbAS erythrocytes inhibit normal parasite development (*1*). This leads to inhibition of parasite transcription and protein expression (*2*). The formation of Maurer’s clefts is impaired due to aberrant actin morphology, which prevent normal protein trafficking (*3*). Binding of parasite proteins which reach the erythrocyte membrane is inhibited by haemichromes, resulting in reduced surface expression of PfEMP (*4*). Reduced PfEMP expression results in less cytoadherence, which results in increased susceptibility to splenic clearance via phagocytosis (*5*). Oxidative stress also results in increased haemachrome formation (*6)*, inducing band 3 mediated senescence and phagocytosis (*7*). Enhanced phagocytosis promotes an immune response (*8*). Increased production of antibodies against parasite proteins further inhibits cytoadherence and amplifies the effect (*9*).

This review has described a number of ways in which erythrocyte disorders may be advantageous to host during malarial infection. It is notable that in several cases multiple mechanisms have been attributed to a single disorder (Table 1). It should also be considered that these effects are not mutually exclusive, although in some cases they may be closely linked. A good example of this is HbAS. In this condition, parasite growth is believed to be inhibited in HbAS cells by the actions of both micro RNAs [70], aberrant actin remodelling [68], and impaired protein trafficking across the parasitophorous vacuole [69]. These mechanisms may also explain the reduced surface expression of PfEMP-1 and associated reduction in cytoadherence [104]. Simultaneously, elevated levels of oxidized haemoglobin in HbAS cells leads to increased deposition of haemichromes on band 3 [122], causing accelerated senescence characterized by band 3 aggregation and binding of naturally occurring antibodies and complement to the erythrocyte surface. The combination of decreased cytoadherence and increased opsonization collectively results in increased phagocytosis of parasitized cells [122]. In addition, increased uptake of parasitized erythrocytes may lead to improved presentation of surface antigens, resulting in the improved antibody response against PfEMP-1 and other parasite surface antigens observed in HbAS individuals [137–139]. Antibodies against PfEMP-1 may further inhibit cytoadherence and facilitate phagocytosis in subsequent infections, which may explain why the protective effect of HbAS increases with age [140, 141] (Figure 1).

It is also plausible that multiple interacting mechanisms involving growth, cytoadherence, and senescence may be the basis for malaria resistance in other erythrocyte disorders. For example, erythrocyte senescence is reported to be accelerated in G6PD deficiency [131], and similar to HbAS cells, increased levels of haemichromes due to oxidative stress are also observed [142]. But it is not yet known if these abnormalities interfere with protein trafficking in the parasite, or concomitantly, its cytoadherent properties. This may also be the case for other disorders where erythrocyte oxidative stress is affected. Interestingly, an early study indicated that parasitized HbAE and HbEE erythrocytes show an increased susceptibility to phagocytosis, however the mechanism behind this was not determined [143]. Given the similarities between HbAE and the other haemoglobinopathies it would be of interest to determine if analogous mechanisms involving senescence are responsible for this phenotype.

## Implications for malaria treatment

The aforementioned mechanisms of malaria resistance conveyed by erythrocyte disorders, particularly those involving invasion, have highlighted several potential parasite proteins suitable for therapeutic targeting. As discussed below, many of these are being developed as potential vaccine targets. An alternate, less conventional approach is the development of host directed therapies (HDTs), which could potentially be designed to mimic the protective effects exhibited by naturally occurring erythrocyte disorders. While targeting the parasite directly is less likely to cause off target effects due to the inherent differences between parasite and human biology, it is hampered by substantial variability between parasite strains, and the remarkable ability of parasites to rapidly mutate and develop drug resistance. HDTs could potentially avoid this problem, as manipulation of the therapeutic target would be outside of direct parasite genetic control. Indeed, erythrocyte disorders have imparted malaria resistance consistently for thousands of years, as opposed to parasite directed antimalarials, some of which have lost effectiveness after as little as 2–3 years of wide-spread use [144]. A caveat to this is that HDTs would be at risk of reproducing the deleterious effects sometimes associated with erythrocyte disorders, although if designed correctly HDTs should theoretically be able to avoid this. In fact, many erythrocyte disorders have little or no clinical symptoms, and yet provide substantial resistance to malaria infection. Additionally, HDTs would only affect the host during short-term treatment regimes, as opposed to genetic mutations, which must be tolerated for life. In this review, four major mechanisms have been described by which erythrocyte disorders can convey malaria resistance. Each of these mechanisms could potentially be exploited therapeutically, either through conventional targeting of parasite proteins, or through HDT.

One strategy is to block erythrocyte receptor interactions required for merozoite invasion, either using vaccines or small molecules. For *P. vivax*, protein structure–function studies have pinpointed the Duffy-antigen binding site of PvDBP to a specific domain called PvDBPII [145], and development of PvDBPII as a vaccine is underway [146]. However, this approach may be challenging due to significant variability in PvDBPII observed amongst different *P. vivax* isolates [147]. More recently, an X-ray crystal structure of the Duffy-PvDBPII complex has been determined, which may help to identify conserved, critical binding sites suitable for therapeutic targeting [148]. Studies with *Plasmodium knowlesi,* which also invades erythrocytes via interactions with the Duffy receptor [149], have shown that a Duffy binding chemokine, melanoma growth stimulating activity (MGSA), can block merozoite invasion by this parasite [150]. Furthermore, as MGSA also binds to type B IL-8 receptor (IL-8RB) and activates neutrophils [151], a mutant form of MGSA was identified that was able to inhibit *P. knowlesi* invasion without cross-reacting with IL-8RB [152]. This result highlights the potential of developing small molecule inhibitors which specifically block the merozoite binding site of the Duffy receptor while avoiding off-target effects, and a screen for these inhibitors has been proposed [153].

Several invasion blocking vaccines for *P. falciparum* are under development. Many are based on the interactions originally identified through the study of naturally occurring erythrocyte disorders, such as the glycophorin deficiencies. Vaccines against the *P. falciparum* merozoite glycophorin binding ligands, PfEBL-1, PfEBA-140 and PfEBA-175 are all under development [22, 154–156]. However, the discovery of glycophorin independent invasion pathways, including CR1-PfRh4 [26] and GYPA-band 3-RhopH3-MSP1 [157], will likely complicate the effectiveness of such approaches. A more ideal vaccine target in this respect may be the basigin-PfRh5 interaction. Antibodies targeting this complex have been shown to completely block erythrocyte invasion by all *P. falciparum* strains tested to date [29, 158, 159]. Preliminary studies have also have indicated that vaccine induced antibodies to PfRh5 inhibit the growth of PfRh5 polymorphic *P. falciparum* strains in vitro [158, 160], and PfRh5 vaccinated Aotus monkeys were protected from experimental infection [161]. In terms of HDTs, antibody blocking of basigin as opposed to PfRh5 has been demonstrated to be more efficacious in preventing invasion [28, 162]. This is possibly due to the fact that merozoite antigens are only exposed for seconds while erythrocyte receptors are continuously exposed, allowing binding to reach equilibrium [162]. Mimicking protective cytoskeletal abnormalities is also an appealing, but probably more challenging approach. However, artificial inhibition of band 3 mobility has already been demonstrated to prevent merozoite invasion [53], opening up the possibility of achieving this in a clinically relevant manner.

The parasite depends on a wide range of host factors in order to facilitate its development and survival within the erythrocyte, and natural protective mechanisms that affect such factors indicate a number of potential targets. In some cases inhibitors for such factors have already been developed, and preclinical efficacy demonstrated. For example, G6PD deficiency is known to reduce malaria susceptibility, and recent chemical screens have identified inhibitors for both human G6PD [163], and *P. falciparum* G6PD [164], with the later demonstrated to reduce parasite growth in vitro. Inhibitors for the host kinase MEK1 have also shown anti-malarial properties, and it has been suggested these compounds may act against the parasite in a host directed manner [85]. Erythrocyte enzymes that are imported by the parasite have also been examined as potential antimalarial targets. Studies have shown that the parasite imports and utilizes the host erythrocyte enzyme Fech [165], and that the parasite-expressed Fech is dispensable [79]. More recently it was demonstrated that parasite growth is reduced in both mouse and human erythrocytes with reduced Fech activity, and that a Fech inhibitor N-methylprotoporphyrin completely prevented parasite growth in vitro [84]. Taken together, this suggests that anti-Fech agents can act against the parasite in a host directed manner. In another example, parasite growth is blocked in erythrocytes treated with an irreversible inhibitor of peroxiredoxin [166]; the parasite normally imports and utilizes host peroxiredoxin-2 during its development [89]. Sotrastaurin is a PKC inhibitor that is currently undergoing Phase II clinical trials for the treatment of psoriasis and in liver transplant [167, 168]. The drug is also highly protective in mice infected with *Plasmodium berghei* and *P. yoelii*, and inhibits *P. falciparum* growth in vivo [87]. Interestingly, no parasite orthologue for the PKC enzyme exists, suggesting the drug may act in a host-directed manner against erythrocyte PKC.

Cytoadherence of infected erythrocytes is another attractive therapeutic target, given it has been implicated in the resistance mechanisms of several erythrocyte disorders. Fucosylated chondroitin sulfate was found to inhibit cytoadherence to human lung endothelial cells by *P. falciparum* infected erythrocytes, including clinical isolates, highlighting its potential therapeutic value [169]. The anthelmintic drug levamisole hydrochloride has been shown to reduce cytoadherence of infected erythrocytes to CD36 [170], however, in a recent randomized controlled trial no clinical benefit was gained when given as an adjuvant to artesunate [171]. This result may indicate that CD36 binding is not detrimental during malaria infection, a notion supported by the fact that the malaria protective condition SAO enhances CD36 binding [107]. In another study, erythrocyte casein kinase II, which is involved in phosphorylation of PfEMP-1, has been pharmacologically inhibited to reduce cytoadherence of infected erythrocytes [172].

Erythrocyte senescence has also proven to be a practical antimalarial target, and compounds designed to accelerate this process have shown potential, with several producing protective effects during the course of malaria infection. These include chlorpromazine [173], paclitaxel [174], the NO synthase inhibitor L-NAME [175], the hormone analogue 16 alpha-bromoepiandrosterone [176], and, indolone-N-oxides [177]. The later are currently undergoing clinical trials for their antimalarial activity. As these compounds induce oxidative stress in the erythrocyte, it would be of interest to determine if they may concurrently inhibit parasite protein trafficking and reduce cytoadherence, as is demonstrated for erythrocyte disorders such as sickle cell trait.

## Conclusions

Studies of erythrocytic genetic disorders associated with host resistance to malaria have arguably been the most important source of knowledge regarding the *Plasmodium*-erythrocyte interaction to date. These studies have not only facilitated the discovery of many of the host-parasite protein interactions involved in merozoite invasion of erythrocytes, but have also provided clues as to what host factors are important for the intraerythrocytic development of the parasite. It is a testament to the complexity of this delicate interaction that even after nearly 70 years of research, entirely new mechanisms for host resistance in conditions such as sickle cell trait, as well as completely novel host-parasite receptor interactions, are being elucidated. Indeed, much about the *Plasmodium*-erythrocyte interaction in erythrocytic genetic disorders remains unclear. As parasite resistance to antimalarials becomes increasingly prevalent, the development of novel therapies will be more important than ever. Research into the mechanisms of resistance provided by erythrocyte disorders is highlighting the key factors required for parasite survival, and the potential for targeting these therapeutically, both through conventional parasite-directed treatments, or through HDT.
